# Theoretical Application of Irreversible (Nonequilibrium) Thermodynamic Principles to Enhance Solute Fluxes across Nanofabricated Hemodialysis Membranes

**DOI:** 10.1155/2012/718085

**Published:** 2012-11-06

**Authors:** Assem Hedayat, Hamdi Elmoselhi, Ahmed Shoker

**Affiliations:** ^1^College of Dentistry, University of Saskatchewan, 105 Wiggins Road, Saskatoon, SK, Canada S7N 5E4; ^2^Saskatchewan Transplant Program, St. Paul's Hospital, University of Saskatchewan, Saskatoon, SK, Canada S7M 0Z9; ^3^Division of Nephrology, Department of Medicine, University of Saskatchewan, 103 Hospital Drive, Saskatoon, SK, Canada S7N 0W8

## Abstract

*Objective*. Nanotechnology has the potential to improve hemodialysis membrane technology. Thus, a major objective is to understand how to enhance toxic solute fluxes across these membranes. The aim of this concept building study is to review the application of irreversible thermodynamic (IT) to solute fluxes. *Methods*. We expanded the application of the Nernst-Planck equation to include the Kedem-Katchalsky equation, pH, membrane thickness, pore size, and electric potential as variables. *Results*. (1) Reducing the membrane's thickness from 25 *μ* m to 25 nm increased the flux of creatinine, *β*_2_-microglobulin, and tumor necrosis factor- *α* (TNF- *α* ) by a thousand times but prevented completely albumin flux, (2) applying an electric potential of 50–400 mV across the membrane enhanced the flux of the respective molecules by 71.167 × 10^−3^, 38.7905 × 10^−8^, and 0.595 × 10^−13^ mol/s, and (3) changing the pH from 7.35 to 7.42 altered the fluxes minimally. *Conclusions*. The results supported an argument to investigate the application of IT to study forces of fluxes across membranes. Reducing the membrane's thickness—together with the application of an electrical potential—qualities achievable by nanotechnology, can enhance the removal of uremic toxins by many folds. However, changing the pH at a specific membrane thickness does not affect the flux significantly.

## 1. Introduction

Irreversible (nonequilibrium) thermodynamics (IT) is a descriptive and powerful tool to delineate the contribution of forces responsible for fluid movements across membranes. Both Soltanieh and Gill [1] and Sievertsen [2] presented excellent reviews summarizing the differences between IT and kinetic transport models. Kedem and Katchalsky [3] stressed that kinetic equations describing volume and solute flow do not fully describe a membrane's physical behavior. They also pointed out to the quantitatively incomparable results of permeability data obtained by different methods. Kedem and Katchalsky resolved this issue by applying IT methods to address membrane transport processes. The principle is to identify the constituent, independent, and elemental processes within the system (diffusion, convection, and so forth…). Then, each process is represented by a set of flux and conjugate force, where there is a relationship between the flux (flow) and the force (free energy gradient) causing it. All these parallel processes of fluxes and conjugate forces can be summed up [4, 5].

Hemodialysis is a life-saving procedure to treat patients with kidney failure. During hemodialysis treatment, the human blood is filtered through a semipermeable membrane to remove the retained toxins because of kidney failure. The principle of hemodialysis is reviewed elsewhere [6–10] and is beyond the scope of this paper. Hemodialysis is an irreversible, nonequilibrium process [11]. As a matter of fact, hemodialysis in equilibrium will not be attractive to professionals in the field, because, at equilibrium, there will be no flow of toxins through the membrane [2]. Numerous kinetic models were developed to describe the flow of uremic toxins through hemodialysis membranes, but little attention was given to IT for the following reasons:

1. Early models of IT were purely diffusive and were missing the convection term although experimentally observed fluxes consisted of both diffusive and convective fluxes. Eventually, a model that contained the convective flux term was developed, to fill the gap in IT models [1].
2. Basic knowledge of membranes characteristics was lacking, and accordingly researchers directed their attention to learn more about membranes' structures and properties such as porosity, pore sizes, tortuosity, permeability, and diffusivity of solutes through them … Knowledge of all these characteristics would have helped to predict the membrane's performance without testing the membrane under actual operating conditions [2]. This prompted researchers to move in this direction.
3. Researchers faced new hurdles. Within the membrane, there are charged walls and pressure-driven processes within capillary spaces, and in hemodialysis membranes, the uremic solute to be filtered has to travel a long distance as compared to its largest dimension [12]. The complex structure of membranes, pore geometry, and the hindered transport of large molecules in liquid-filled pores led research in the direction of transport kinetics [13, 14].

In models based on IT, the membrane is treated as a black box where processes take place in it slowly under close to equilibrium conditions, and with no knowledge of the process by which the solutes migrate through the membrane [1]. The difference between IT models and conventional kinetic models with regards to approaching solute flux can be summarized as follows: IT models are unsusceptible to neither pressure nor concentration [1]. Kinetic models, on the other hand, are governed by the solute clearance of the dialyzer, as well as the rates of toxins being produced and their concentrations [15].

In most cases, the fluxes are not linearly dependent on the driving forces like concentration and pressure. Thus, IT avoids going into details of solving differential equations within the membrane. For example, the Kedem-Katchalsky model is relatively insensitive to both the concentration and pressure driving forces. Numerical coefficients in IT models are not functions of the driving forces. Thus, less experimentation to measure these coefficients is necessary. Some models are better than others based on the sensitivity of the coefficients to the driving forces. Nonequilibrium of filtration processes is a reality. Research showed that it would take anywhere between 16 and 48 hours for the diffusion of salt in a 7.5-micron thick membrane to reach equilibrium [1].

Nanofabricated membranes are a new class of membranes that have a great potential in effectively separating neutral or charged solutes. These membranes are characterized by structural parameters such as membrane thickness, pore radius, and electrical properties [16].

One of the basic advantages of nanofabricated hemodialysis membranes and the membranes currently used is that the former may be produced in thicknesses as fine as 25 nm, which is 1000 times thinner. Nanotechnology can also produce nanopores that allow the selective removal of uremic toxins, while retaining beneficial, large molecules, such as albumin, from passing through. As will be shown in the next sections, a 1000 times reduction in thickness can translate into a 1000 times increase in flux of a uremic molecule. Also, the thickness of the nanofabricated membrane is comparable in its dimension to that of a uremic toxin molecule. 

So far, experimentation with nanofabricated membranes, built for hemodialysis application, focused on flat-plate designs rather than the hollow fiber ones. In hollow fiber filters, the structure of the polymeric membrane is characterized by its tortuous porosity and wide pore size distribution [17]. In comparison, flat-plate filters have a controlled pore size dispersion but are made of silicon [18] and aluminum oxide [19], which are brittle materials. The reason for selecting Si and Al_2_O_3_ for the flat-plate design is that their nanofabrication techniques are advanced and well established as compared to other materials. The feasibility of producing nanofabricated hemodialysis membranes, and applying them in practice, will depend on the advancement of nanofabricated techniques that can be applied to materials with better mechanical properties than Si and Al_2_O_3_.

## 2. Results

All symbols are listed and defined in the abbreviation list.

### 2.1. Including pH in the Extended Nernst-Planck Equation

The proton motive force is a gradient affecting transport across membranes. Consider the following reaction where two Hydrogen ions (protons) are reduced:

(1) $$\begin{array}{c} 2{\text{H}}^{+}+2{\text{e}}^{-}={\text{H}}_{2} \end{array}$$

H_2_: Δ*G* ^0^ = 0 (by definition)
H^+^: Δ*G* ^0^ = 0 (by convention)
(2) $$\begin{array}{c} \xi^{0}=\frac{-\Delta G^{0}}{zF}=0, \end{array}$$

where, *ξ* ^0^ is the standard potential. Thus, the potential difference becomes

(3) $$\begin{array}{c} \xi -\xi^{0}=2.303\frac{RT}{zF}\log\frac{{\left[{\text{H}}^{+}\right]}^{2}}{P_{{\text{H}}_{2}}}. \end{array}$$

And since pH = −log⁡  [H^+^]

(4) $$\begin{array}{c} \xi =-2.303\frac{RT}{zF}\left(2\right)\text{pH}-2.303\frac{RT}{zF}\log P_{{\text{H}}_{2}}, \end{array}$$

(5) ξDeffACmxzFRT=−4.606DeffAxCmpH−2.303DeffAxCmlog⁡PH2,

where *P*_H_2__ is the partial pressure of H_2_ at 37°C.

Thus, we expanded the Nernst-Planck equation as follows:

(6) J=−DoAKdiff(dCdx)−(DeffACmzFRT)(dVdx)−(ξDeffACmzFxRT)+KconvACmJv.

Substituting (5) into (6), we get

(7) J=−DoAKdiff(dCdx)−(DeffACmzFRT)(dVdx)−(DeffACmx)(−4.606pH−2.303log⁡PH2)+KconvACmJv.

Note that the negative sign for *J*_diff_ and *J*_electromigr_ indicates that *J* is positive when the solutes mobility is down a gradient. In other words, the negative sign cancels the negative gradient along the direction of positive flux. Thus, all quantities *J*_diff_ + *J*_electromigr  _ + *J*_proton  motive  force_ + *J*_conv_ can have a synergistic effect. 

### 2.2. Promoting Fluxes by Applying an Electric Potential to Existing Membranes

The applied electric potential enhanced the fluxes of selected uremic toxins as follows:

For creatinine:
(8) $$\begin{array}{c} \left(\frac{J_{\text{electromigr}}}{z}\right)=203.34\times 10^{-6}dV. \end{array}$$
For *β*_2_-microglobulin:
(9) $$\begin{array}{c} \left(\frac{J_{\text{electromigr}}}{z}\right)=110.83\times 10^{-11}dV. \end{array}$$
For tumor necrosis factor–*α*:
(10) $$\begin{array}{c} \left(\frac{J_{\text{electromigr}}}{z}\right)=1.7\times 10^{-16}dV. \end{array}$$
And, for albumin:
(11) $$\begin{array}{c} \left(\frac{J_{\text{electromigr}}}{z}\right)=0, \end{array}$$

where *J*_electromigr_ is in mol/s, and V is in volts.

### 2.3. Extending the Nernst-Planck Equation to Include the Kedem-Katchalsky Equation

The Nernst-Planck equation can be extended further to include the Kedem-Katchalsky equation

(12) $$\begin{array}{c} \;J_{p}=L_{p}\;A\;\left(\Delta P+\Delta \pi \right), \end{array}$$

where, *J*_*p*_ is the flux contributed by ultrafiltration, *A* is the area of membrane (m^2^), *L*_*p*_ Is hydraulic permeability of the membrane for water, that is, the volumetric flow rate of water per unit area of membrane per unit pressure gradient (ml/min/m^2^/mmHg), ΔP is the hydraulic pressure gradient from blood path to dialysis fluid path (mmHg), and Δ*π* is the osmotic pressure gradient from blood path to dialysis fluid path (~19mmHg)

Thus, the extended Nernst-Plank equation can be written as:

(13) J=−DeffAKdiff(dCdx)−(DeffACmzFRT)(dVdx)−(DeffACmx)(−4.606pH−2.303log⁡PH2)+KconvACmJv+ΩLpA(ΔP+Δπ).

**Figure 1:**
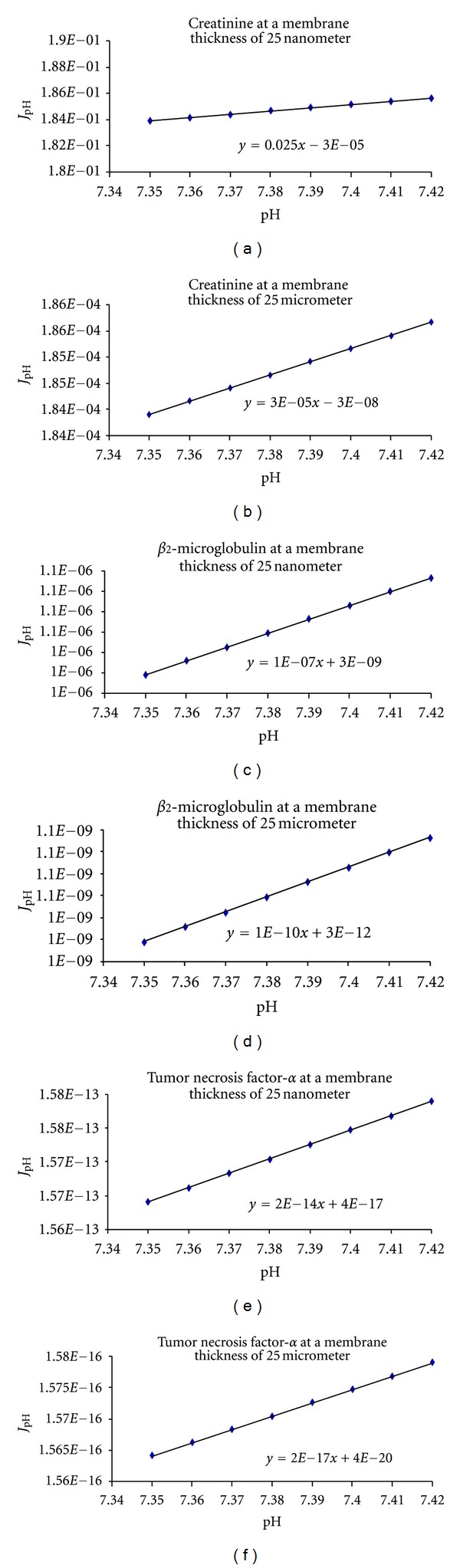
Effect of pH and membrane thickness on solute flux as calculated from  *J* = −*D*_eff_ *AK*_diff_(*dC*/*dx*) − (*D*_eff_ *AC*_*m*_ *zF*/*RT*)(*dV*/*dx*) − (*D*_eff_ *AC*_*m*_/*x*)(−4.606 pH − 2.303log⁡*P*_*H*_2__) + *K*_conv_ *AC*_*m*_ *J*_*v*_. Creatinine molecular volume 110.55 Å_3_, *β*_2_-microglobulin molecular volume 14,514.81 Å_3_, and tumor necrosis factor-*α* molecular volume 31,979.13 Å_3_ (Fluxes are expressed as mol/s; *P* < 0.0001 and *R* ^2^ > 0.95 in all instances).

**Figure 2:**
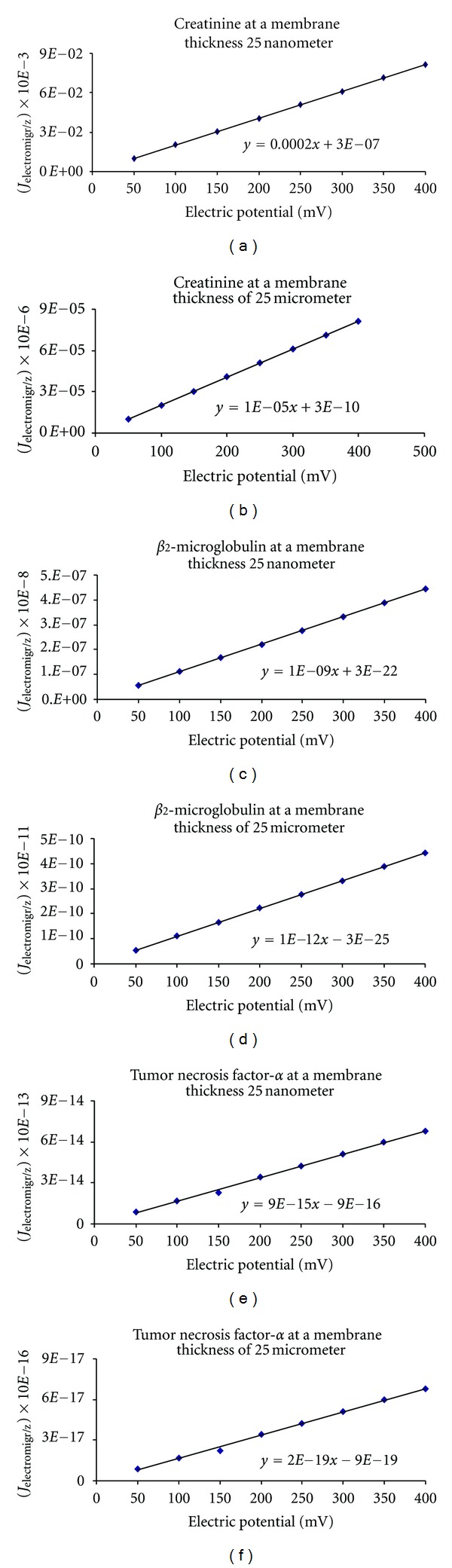
Effect of electrical potential and membrane thickness on solute flux as calculated from *J* = −*D*_eff_ *AK*_diff_(*dC*/*dx*) − (*D*_eff_ *AC*_*m*_ *zF*/*RT*)(*dV*/*dx*) − (*D*_eff_ *AC*_*m*_/*x*)(−4.606 pH − 2.303log⁡*P*_*H*_2__) + *K*_conv_ *AC*_*m*_ *J*_*v*_. Creatinine molecular volume 110.55 Å_3_, *β*_2_-microglobulin molecular volume 14,514.81 Å_3_ and tumor necrosis factor-*α* molecular volume 31,979.13 Å_3_. (Fluxes are expressed as mol/s; *P* < 0.0001 and *R* ^2^ > 0.95 in all instances; Note the major impact of mV and thickness on fluxes.)

Note that (12) was modified by multiplying it by a solute concentration parameter, Ω, needed to balance the units on both sides of (13). We applied the above equations to illustrate the dependency of fluxes on pH, electric potential, and membrane thickness for selected uremic toxins: creatinine, *β*_2_-microglobulin, and tumor necrosis factor-*α*.  Figure 1 shows the effect of pH on solute flux. It is illustrated in the figure the little effect that pH has over the flux at specific thicknesses. Also, notice that albumin has no flux, which indicates that it is not passing through.  Figure 2 illustrates the effect of the electric potential and membrane thickness on solute flux. In the same figure, it is shown how the application of the electric potential increases the flux for these molecules. Both Figures 1 and 2 illustrate that for a nanofabricated membrane, 25 nm thick, a reduction in membrane thickness increases the flux significantly. Notice that the flux increased a 1000 times when the thickness of the membrane was reduced 1000 times. 

## 3. Discussion

### 3.1. Application of IT to Nanofabricated Membranes

 During hemodialysis, as expressed by IT terms, entropy is generated per unit volume of the membrane as a result of a nonequilibrium process at the rate of *d*σ**/*dt*. Multiple forces act on the species in the system simultaneously leading to simultaneous fluxes. We get sets of conjugate forces and fluxes [4] which can be represented as follows:

(14) $$\begin{array}{c} T\sigma =\sum_{i}J_{i}X_{i}=-\Delta G=-\sum_{i}N_{i}\Delta \mu_{i}, \end{array}$$

where *T* is the temperature, *σ* is the entropy (J/mole), *t* is the time, *J*_*i*_ is the diffusion flux of solute species(*i*) = −*D*_*i*_(*dc*_*i*_/*dz*), *D*_*i*_ is the diffusivity, *dc*_*i*_/*dz* is the concentration gradient, ΔG is the Gibbs free energy, *N*_*i*_ is the molar flux of solute *i*, and Δ*μ*_*i*_ is the chemical potential of solute (*i*) [11].

For a favorable filtration process of a uremic toxin across a hemodialysis membrane, the change of Gibb's free energy, ΔG, of the transported species has to be negative. The more negative the ΔG is, the more favorable the transport will proceed. If ΔG is positive or zero, no transport will take place.

IT deals with a hemodialysis membrane as a surface of specific surface area and thickness. A nanofabricated membrane has a higher surface area to volume ratio than a synthetic hemodialysis membrane. All fluxes derived through IT are directly proportional to the surface area of the membrane and inversely proportional to its thickness. 

### 3.2. Promoting Fluxes by Applying an Electric Potential to Existing Membranes

If we examine the extended Nernst-Planck equation [20], we find that the flux is a function of the concentration and electric potential gradients. So, if we nanofabricate a membrane 25 nm thick, the flux will be 3 orders of magnitude greater than if hemodialysis was pursued using a 25-micron thick hemodialysis membrane. Consider

(15) J=−D0AKdiff(dCmdx)−(DeffACmzFRT)(dVdx)+KconvACmJv   [20],

where *J* is the molecular flux (mol/s), *A* is the membrane's surface area (m^2^), *C*_*m*_ is the solute's concentration inside the membrane (mol/m^3^), *z* is the valence, *F* is Faraday's constant (Coulomb/mol), *R* is the gas constant (J/mole · K), *T* is the temperature (K), (*dV*/*dx*) is the electric potential difference across the membrane, and *J*_*v*_ is the parabolic fluid velocity (m/s).

The equation incorporates the hindrance factors *K*_diff_ and *K*_conv_ for diffusion and convection, respectively [20]. These hindrances are attributed to solute-wall hydrodynamic interactions [21]. It is worth mentioning that, due to the randomized shapes of biomolecules, the diffusivities of molecules through the membrane will vary significantly [22].

In spite of the increasing use of hemodialysis, the sieving and transport mechanisms are not fully understood, and the solute retention models are not accurate. It is essential to understand both the transport mechanisms and the sieving process so we can develop better membranes [2].

By controlling the structure of the membranes with respect to porosity, permeability, diffusivity, and so forth, we can produce more accurate kinetic models that can explain the transport mechanism inside the membrane and the sieving process. New design parameters such as molecular volume, shape, electric charges, and molecular conformity will dominate sieving parameters. 

With the emerging nanotechnology and our capability to nanofabricate thinner hemodialysis membranes with nanopores of unique geometrical configurations and periodicity, nonequilibrium (irreversible) thermodynamics will play a larger role in modeling the fluxes of uremic toxins through the nanofabricated membranes. In currently used polymeric membranes, with a thickness of 25 microns, uremic toxin molecules travel a much longer distance as compared to their maximum diameters. This is in contrast to traveling only a few times their thickness through an ultrathin nanofabricated membrane. 

Nanofabricated membranes technology can take advantage of creating an electric potential difference across the hemodialysis membranes. The membranes can be made conductive by applying an atomic metallic layer on its surface. Thus, there is an additional driving force in play, which is the electrical potential gradient. The process by which the molecules/ionic solutes transport under this gradient is known as electromigration [23]. Filtration across the nanofabricated membranes will also be governed by solute concentration (diffusion), pressure (convection), pore size, molecular charge, and surface tension. It is worth mentioning that a deviation from the pore geometry in synthetic membranes may lead to hindrance in solute passage as a result of changes in hydraulic permeability [24–26]. Experimentation with the conductive layer thickness, material deposited, and whether direct or alternating current which will be applied to the membranes is necessary to determine the optimal voltage and current needed to enhance the clearance of uremic toxins.

Achieving optimal electrical potential on the surface of a nanofabricated membrane will require extensive research, especially in the area of solute flux and hemocompatibility. Uremic toxins exhibit different electrical characteristics. While urea has no net charge [27], creatinine has a net positive charge [28], and interleukin-6 exhibits positive surface charges at different sites and negative surface charges at others [29]. Thus, extensive research should be pursued to set the standard for the optimal potential required to yield the most-efficient solute flux. Research proved that membranes with a controlled potential proved to me more biocompatible, and yielded improved clearance of small sized uremic toxins [30]. 

In the 1990's, the hemodialysis membrane AN69, which was made of polyacrylonitrile (PAN), was regarded as the most biocompatible membrane. AN69 adsorbed positively charged proteins as its surface was negatively charged. The membrane promoted the filtration of *β*2-microglobulin and complement activation, but, at the same time, it adsorbed the higher molecular weight kininogen. This resulted in contact activation and an elevated surplus blood volume [31].

Hemoincompatibility has long been considered as a main problem in dialysis treatment [32–35]. It causes inflammation in dialysis patients and therefore affects their morbidity and mortality. For example, the cardiac effects of chronic inflammation in dialysis patients are well recognized. The prevalence of cardiac disease is high in uremic patients just beginning dialysis and even more so in cases of lateral referral. The excessive risk of cardiac diseases in chronic uremic patients is in part due to dialysis-related bioincompatibility [36].

Nanofabricating membrane technology can bring the main driving forces of molecular sieving into synergy. These driving forces are diffusion (concentration gradient), convection (pressure gradient), electromigration (electric potential gradient), and proton motive force (pH and membrane potential gradients). Synthetic membranes currently used in hemodialysis are mainly polymeric and are characterized by their low efficiency. This low efficiency is attributed to the dissynergistic effect between the driving forces of filtration, namely, diffusion and convection. Diffusion is driven by a concentration gradient, and convection is driven by a pressure gradient. The conjoint effect of these two molecular transport mechanisms across the synthetic membrane is less than the sum of their solitary effects combined. That is why they are referred to as dissynergistic. This is opposite to other mechanisms where the conjoint effect of processes is greater than the sum of their solitary effects combined with synergistic effect [37]. We use the term “dis-synergistic” as the opposite of synergistic instead of the term “antagonistic” because the latter is not accurate in this context.

The flux rate is directly proportional to the rate of fluid movement across the membrane [38]. Nanofabrication reduces the probability of a flexible molecule that enters a pore that has a smaller diameter than its radius of gyration, and the molecule will try to stretch itself by exerting energy to overcome an entropic energy barrier. It can get trapped at the pore's interface. This is known as entropic trapping. This can occur even if the pore's size is much larger than the backbone radius of the flexible molecule [39]. Also, applied fields such as the electrical fields on the surface of the membrane can add to the complexity of filtration. For example, some molecules can change shape in the presence of electrical fields [40].

The proton motive force is another gradient effecting transport across membranes [41–44]. In reviewing the literature, we noticed that the pH has been neglected from all hemodialysis membrane models and was, therefore, added in our calculations. 

### 3.3. Effect of Membrane Thickness on Electromigration Flux

The objective of nanofabricating a hemodialysis membrane is to increase the flux of uremic toxins particularly the middle molecules. However, large, beneficial molecules such as albumin should not pass through. As (15) clearly indicates, the flux will increase with the reduction in membrane thickness. But at the same time, the membrane has to be selective in its removal, or else albumin will pass through. 

## 4. Concise Methods

For each molecule, the volume of its crystal lattice, the number of molecules per lattice, and the solvent content percent were calculated using classical crystallographic equations. To estimate the contribution of the applied electric potential to the flux, we used the following equation:

(16) $$\begin{array}{c} J_{\text{electromigr}}=\left(\frac{D_{\text{eff}}AC_{m}zF}{RT}\right)\left(\frac{dV}{dx}\right). \end{array}$$

To determine the maximum radius of a solute, Bowen et al. [16] and Sun et al. [45] used the following equation:

(17) $$\begin{array}{c} \log r=\;-1.3363+0.395\log\;\left(\text{MW}\right)\;\;\left[16\right], \end{array}$$

where the molecular weight (MW) is expressed in Daltons, and (*r*) in nm. We too applied (17), and according to these calculations, the cutoff molecular diameter was determined at 6.04, which corresponds to Interleukin-1*β*. From the design point of view, there is thus room for improvement to establish sound principles to manufacture efficient hemodialysis membranes using nanotechnology.

To support the concept that the application of an electric potential across a membrane can enhance the clearance of uremic toxins during hemodialysis, we calculated *J*_electromigr_ for three molecules as a function of voltage. The selected molecules are creatinine (molecular volume of 110.55 Å^3,^ molecular weight of 113 Da), *β*_2_-microglobulin (molecular volume of 14,514.81 Å^3^, molecular weight of 11,800 Da), and tumor necrosis factor-*α* (molecular volume of 31,979.13 Å^3^, and molecular weight of 26,000 Da). The valence is only known for small molecules like creatinine. For larger molecules, however, the valence depends on the pH of the medium and is not reported accurately in the literature for most uremic toxins. Thus *J*_electromigr_ was estimated per net charge (*z*). We also applied our calculations to albumin (molecular weight of 69,000 Da) to ensure that it will not go through.

To calculate *C*_*m*_, we used AN69 as the reference membrane with 80% porosity, a unit surface area of 1 m^2^, and a thickness of 25 microns. The concentration of each solute was determined from the sieving coefficients (*S*) illustration for hemofiltration membranes (such as AN69) as a function of molecular weights [46]. The sieving coefficient is defined as the ratio of the solute concentration in the membrane, *C*_*m*_, to the bulk concentration of the solute prior to filtration, *C*_bulk_. This can be represented as

(18) $$\begin{array}{c} C_{m}=SC_{\text{bulk}} \end{array}$$

(see [47]).  Table 1 summarizes the normal concentration of selected uremic toxins in the blood as well as their sieving coefficients. The free diffusivity of the molecules (*D*_0_) was calculated using the following equation:

(19) $$\begin{array}{c} D_{0}=\frac{13.26\times 10^{-5}}{\eta^{1.4}\times V_{M}^{0.589}} \end{array}$$

(see [48, 49]), where *η* is the viscosity of water at 37°C, and V_M_ is the molecular volume. 

And, *D*_eff_ was calculated as follows:

(20) $$\begin{array}{c} D_{\text{eff}}=D_{o}K_{\text{diff}}, \end{array}$$

where *D*_eff_ is the effective diffusion coefficient (m^2^/s). 

 These calculations were compared with the calculations of *D*_eff_ for a nanofabricated membrane 25 nm thick, where the *K*_diff_ was calculated as follows.

If the solute passing through a pore has a radius “*r*,” then depending on the pore geometry, we can assign “*b*” as the radius of the cylindrical pore, or 1/2 as the width of a pore. 

We represent the relative solute size *λ* as the ratio *r*/*b*.

Thus, in diffusion:

(21) $$\begin{array}{c} K_{\text{diff}}\to 1\;\;\text{as}\;\;\lambda \to 0,\\ K_{\text{diff}}\to 0\;\;\text{as}\;\;\lambda \to 1,\\ K_{\text{diff}}=1.0-2.3\lambda +1.154\lambda^{2}+0.224\lambda^{3} \end{array}$$

(see [20, 50]). But, whether *K*_diff_ disappears, as *λ* → 1, depends on the pore's shape [21]. 

There are limitations to this work. Proof of concept in a practical experiment, and future clinical study is needed to confirm the results. Rapid solute removal has obvious disadvantages. The emphasis of this work is to present an initial theoretical framework for future nanobased membrane design.

We reviewed the application of IT to study modifiable factors that can be achieved by nanotechnology to enhance solute fluxes. The results are encouraging in that (1) it is likely that, through nanofabrication, we can synergize the driving forces of hemodialysis. With current membranes, diffusion and convection are dis-synergistic. (2) The application of an electric field to the membrane can give rise to an electric driving force, which will overwhelm diffusion and convection and promote synergy between all driving forces of hemodialysis. And (3) thinner membranes will likely improve solute fluxes.

## Figures and Tables

**Table 1:** Normal concentration of selected uremic toxin molecules in the blood and sieving coefficient.

Molecule	Normal concentration*	Sieving coefficient**
Creatinine	10.2 mg/L [51]	1.0
*β*_2_-microglobulin	<2.0 mg/L [52]	0.35
Tumor necrosis factor-*α*	13.3 ± 3.0 ng/L [52]	0.2

*Used as *C*_bulk_.

**Determined from sieving coefficient versus molecular weight illustration [46].

## Abbreviation

H^+^: Hydrogen ion (proton)
H_2_: Hydrogen molecule
e^−^: Electron
ΔG^o^: Standard Gibbs free energy
*ξ* ^o^: Standard electrode potential
*z*: Valence
*F*: Faraday's constant
*ξ*: Electrode potential
*p*_H_2__: Partial pressure of hydrogen
*R*: Gas constant
*D*_eff_: Effective diffusivity
*C*_*m*_: Solute's concentration inside the membrane
*A*: Area of the membrane
*x*: Thickness of the membrane
*D*_o_: Diffusion coefficient
*J*: Solute flux
*dC*/*dx*: Concentration gradient across the membrane
*T*: Temperature
*dV*/*dx*: Electric potential difference across the membrane
*k*_diff_: Diffusion hindrance
*k*_conv_: Convection hindrance
*J*_*v*_: Parabolic fluid velocity
*J*_diff_: Solute flux by diffusion
*J*_electromigr_: Solute flux by electromigration
*J*_proton motive force_: Solute flux by the proton motive force
*J*_conv_: Solute flux by convection
*J*_*p*_: Solute flux by ultrafiltration
*L*_*p*_: Hydraulic permeability of the membrane for water, that is, the volumetric flow rate of water per unit area of membrane per unit pressure gradient (mL/min/m^2^/mmHg)
Δ*P*: Hydraulic pressure gradient from blood path to dialysis fluid path
Δ*π*: Osmotic pressure gradient from blood path to dialysis fluid path
*Ω*: Solute concentration parameter
*σ*: Entropy
*t*: Time
*J*_*i*_: Diffusion flux of solute species (*i*) = −*D*_*i*_(*dc*_*i*_/*dz*)
*D*_*i*_: Diffusivity of solute species (*i*)
*dc*_*i*_/*dz*: Concentration gradient
Δ*G*: Gibbs free energy
*N*_*i*_: Molar flux of solute (*i*)
Δ*μ*_*i*_: Chemical potential of solute (*i*)
*r*: Molecular radius
*S*: Sieving coefficient
*C*_bulk_: Solute concentration in the bulk plasma water
*η*: Viscosity of water at 37°C
*V*_*M*_: Molecular volume
*b*: Radius of a cylindrical pore
*λ*: Ratio *r*/*b*.
